# Navigating surgical anatomy of the Denonvilliers’ fascia and dissection planes of the anterior mesorectum with a cadaveric simulation model

**DOI:** 10.1007/s13304-022-01252-2

**Published:** 2022-03-14

**Authors:** María García-Gausí, Juan García-Armengol, Gianluca Pellino, Claudia Mulas, José V. Roig, Alvaro García-Granero, David Moro, Alfonso Valverde

**Affiliations:** 1 Surgical Anatomy Unit, Department of Human Anatomy and Embryology, Faculty of Medicine and Dentistry, University of Valencia, Avenida Blasco Ibañez, 15, 46010 Valencia, Spain; 2 Coloproctology Unit, European Center for Colorectal Surgery, Vithas Valencia 9 of October Hospital, Valencia, Spain; 3 Department of General and Digestive Surgery, Punta de Europa Hospital, Algeciras, Spain; 4 Department of Advanced Medical and Surgical Sciences, Università degli Studi della Campania “Luigi Vanvitelli”, Naples, Italy; 5 Coloproctology Unit, Department of General and Digestive Surgery, University General Hospital Consortium, Valencia, Spain; 6 Coloproctology Unit, Department of General and Digestive Surgery, Son Espases University Hospital, Palma, Spain; 7 Coloproctology Unit, Department of General and Digestive Surgery, Clinic University Hospital, Valencia, Spain

**Keywords:** Total meso-rectal excision, Denonvilliers’ fascia, Rectal cancer, Surgical anatomy

## Abstract

Anterior dissection of the rectum in the male pelvis represents one of the most complex phases of total meso-rectal excision. However, the possible existence of different anatomical planes is controversial and the exact anatomical topography of Denonvilliers’ fascia is still debated. The aim of the study is to accurately define in a cadaveric simulation model the existence and boundaries of Denonvilliers’ fascia, identifying the anatomical planes suitable for surgical dissection. The pelvises of 31 formalin-preserved male cadavers were dissected. Careful and detailed dissection was carried out to visualize the anatomical structures and the potential dissection planes, simulating an anterior meso-rectum dissection. Denonvilliers’ fascia was identified in 100% of the pelvises, as a single-layer fascia that originates from the peritoneal reflection and descends until its firm adhesion to the prostate capsule. The fascia divides the space providing an anterior and a posterior plane. Anteriorly to the fascia, during the caudal dissection, its firm adhesion to the prostate capsule forces to section it sharply. The cadaveric simulation model allowed an accurate description of Denonvilliers’ fascia, defining several planes for anterior dissection of the meso-rectum.

## Introduction

In 1836, Charles-Pierre Denonvilliers (1808–1872) described a clear and distinct membranous layer in the male pelvis, behind the prostate and between the seminal vesicles and the rectum, which he named “prostate-peritoneal membrane”. Originating from the anterior peritoneal reflection, it joins the prostatic fascia, separating the rectum from the bladder, seminal vesicles, and the prostate [1]. However, despite this clear initial description, there are still certain discrepancies regarding its embryological origin, composition, exact anatomical topography, and whether a similar “standalone” structure can be found in women [2–5].

Identifying the Denonvilliers’ fascia during in vivo surgery is not straightforward, as well as visualizing its exact relationships with the *fascia recti* and the neurovascular bundles. Of note, most of the parasympathetic nerve lesions occur in this area during the deep dissection of the extra-peritoneal rectum. The proximity of the autonomic nerves and the oncological importance of achieving complete local control of the disease, raise concerns about the correct dissection level to obtain the best oncological and functional results.

For these reasons, anterior dissection of the rectum in the male pelvis represents one of the most complex parts of the total meso-rectal excision (TME) procedure for rectal cancer. The possible existence of different anatomical planes is still controversial, but it could be crucial for optimal oncological resection and, at the same time, important to avoid injuries to pelvic autonomic innervation [3].

The primary aim of the current study was to define precisely in a cadaveric simulation model the existence and boundaries of the Denonvilliers’ fascia in male pelvises, as well as the possible anatomical planes that can be generated in connection with it, suitable for surgical resection of the rectum.

## Method

A descriptive anatomical study was performed using the pelvises of male adult cadavers, who were not affected by proctological disease and did not suffer any surgical traumas at the level of the rectum, bladder, seminal vesicles, and prostate. The male cadaveric pelvic specimens were not used for the purpose of the study if there were difficulties in the dissection of said area due to anatomical variants, or if there were issues with tissues preservation after embalming and handling. The current study has been carried out at the Department of Human Anatomy and Embryology (Faculty of Medicine and Dentistry) of the University of Valencia after obtaining the approval of the ethical committee.

**Fig. 1:**
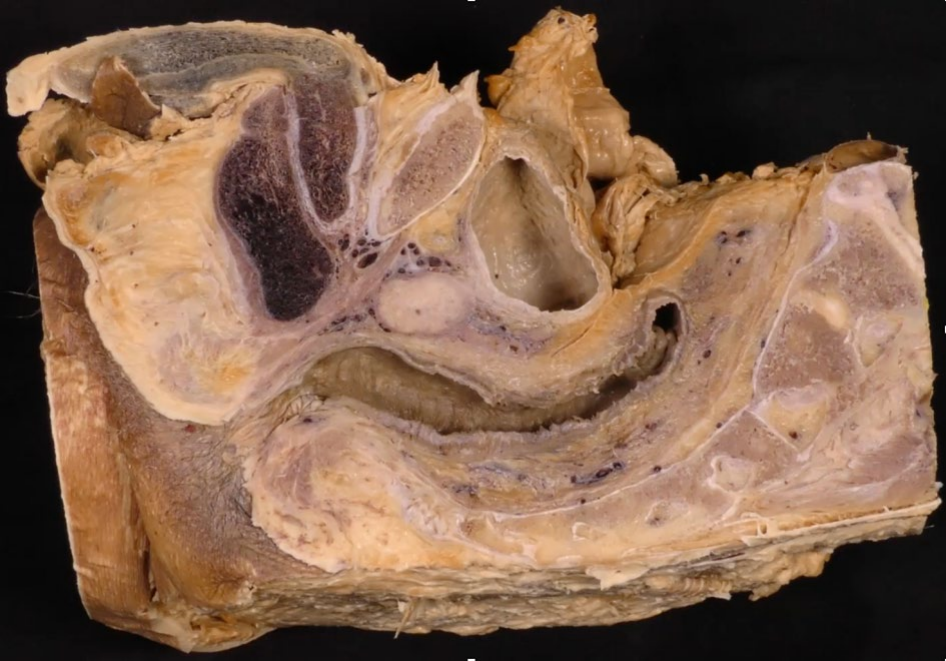
Male pelvis sectioned in a middle sagittal plane

For this study, the pelvises of 31 formalin-preserved male cadavers were dissected. All the included specimens were sectioned along a midsagittal plane, at the level of the middle axis of the anal canal, to allow a correct visualization of the anatomical pelvic structures (Fig. 1). Handling, cadaver selection, and preparation of the specimen have been previously detailed [5].

Once the preparation was completed, careful and detailed dissection of the perirectal structures was carried out by colorectal surgeons with extensive expertise in applied colorectal anatomy, and with the collaboration of human anatomists. Attention was paid to visualize the potential anatomical dissection planes during the cadaveric simulation model of TME. Rectal dissection was started posteriorly, by completely dissecting the retro-rectal space, and it was continued on the lateral portion of the mesorectum, leaving the anterior dissection (focus of the current study) as the last phase.

## Results

**Fig. 2:**
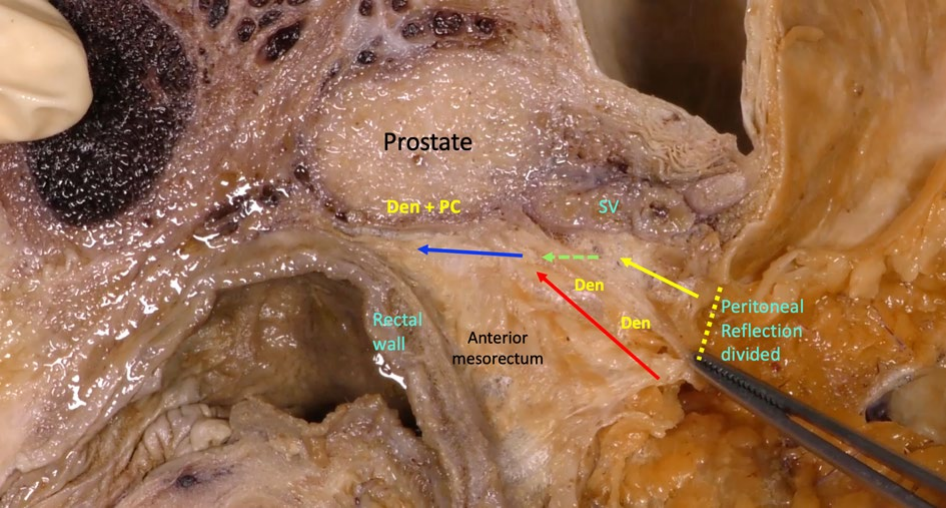
Gentle traction-and-countertraction maneuvers of the space below the anterior peritoneal reflection. Denonvilliers’ fascia (*Den*) descends from the peritoneal reflection to adhere firmly to the prostate capsule (*Den + PC*). Red arrow: Cranial loose areolar plane behind Denonvilliers’ fascia. Yellow arrow: Cranial loose areolar plane in front of Denonvilliers’ fascia and behind seminal vesicles (*SV*). Green arrow: Division of Denonvilliers’ fascia. Blue arrow: Caudal loose areolar plane behind the confluence of Denonvilliers’ fascia and prostate capsule (*Den + PC*) (colour figure online)

**Fig. 3:**
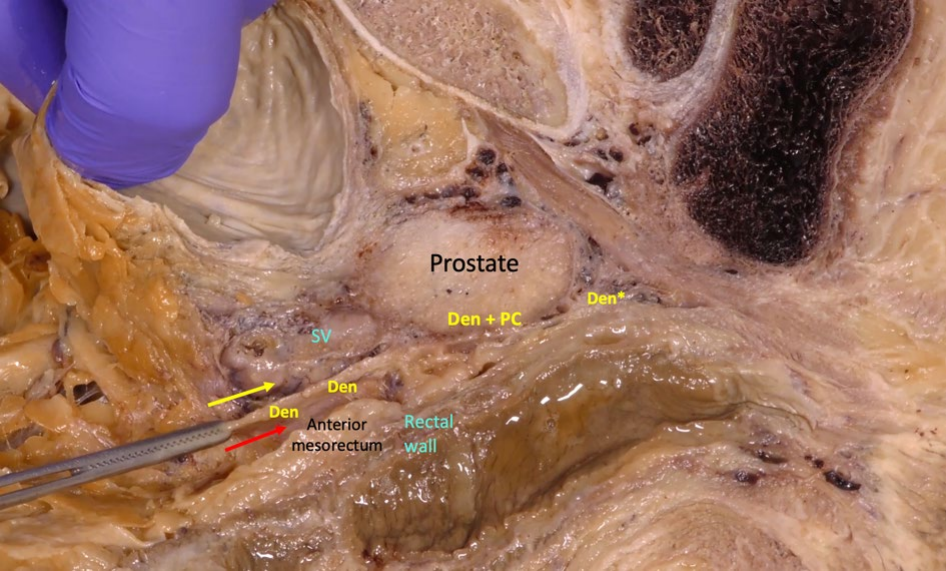
After cranial anterior dissection of loose areolar tissue, forceps make a traction of Denonvilliers’ fascia like a single and consistent layer that offers a posterior plane with the anterior mesorectum (red arrow), and an anterior plane (yellow arrow) in front of said fascia with the seminal vesicles (*SV*). Immediately below the seminal vesicles, the fascia adheres firmly to the prostate capsule in all its extension (*Den + PC*). Den*: Denonvilliers’ fascia extends downward to reach the level of the urogenital diaphragm (colour figure online)

After the complete dissection of the posterior and lateral mesorectum, the space below the anterior peritoneal reflection, between the rectal wall and the seminal vesicles and the prostate, is opened, using gentle traction-and-countertraction maneuvers. This space mainly contains loose areolar tissue that allows for an easy dissection distally. After crossing it in its most cranial portion, a single-layer fascia that originates from the peritoneal reflection is visualized, which descends approaching the seminal vesicles. This fascia offers a posterior plane of loose areolar dissection with the anterior mesorectum, and an anterior plane is identified in front of said fascia with the seminal vesicles. Immediately below the seminal vesicles, the fascia adheres firmly to the prostate capsule in all its extension, and it extends downward to reach the level of the urogenital diaphragm. This fascia, which is consistent with the one originally described by Denonvillier [1], can be identified in 100% of the pelvises, and its route offers different avascular anatomical planes of loose areolar tissue for the surgical dissection of the anterior mesorectum (Figs. 2 and 3).

**Fig. 4:**
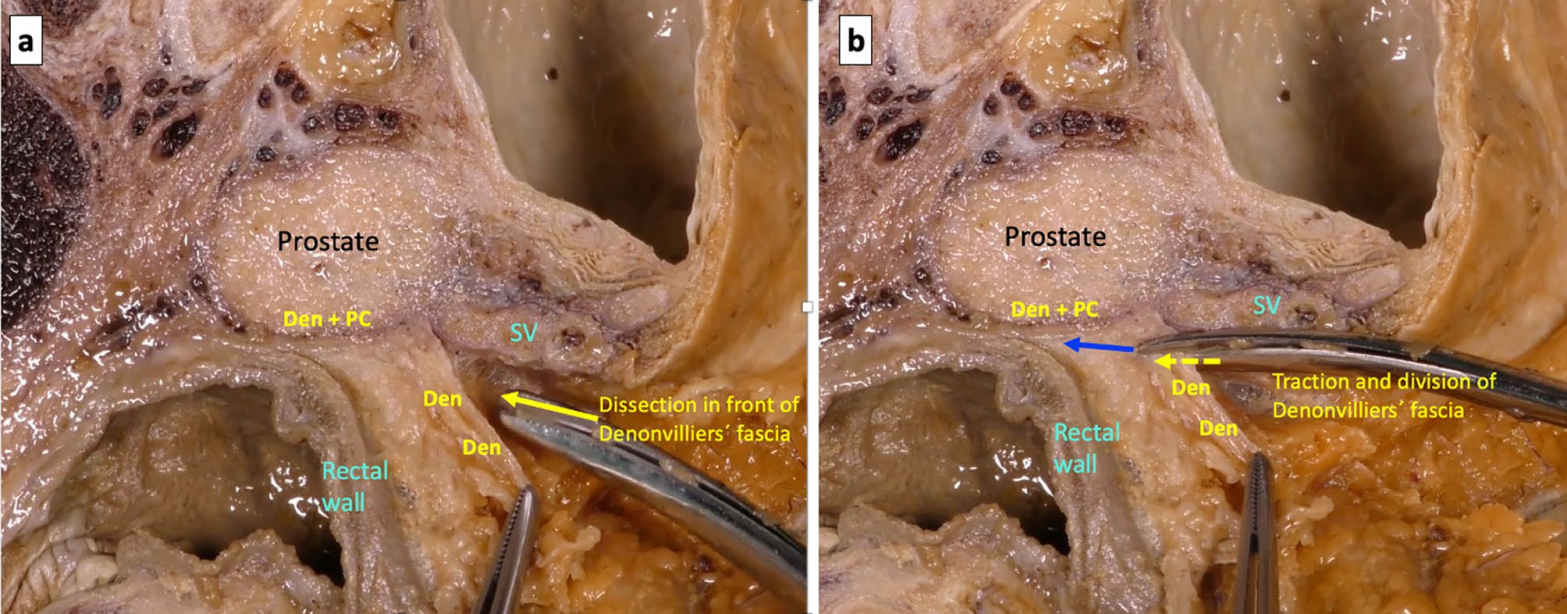
**a** After division of the anterior peritoneal reflection, the backward pressure of the rectum facilitates the cranial loose areolar dissection in front of Denonvilliers’ fascia. **b** Denonvilliers’ fascia must be divided to continue the anterior dissection caudally

**Fig. 5:**
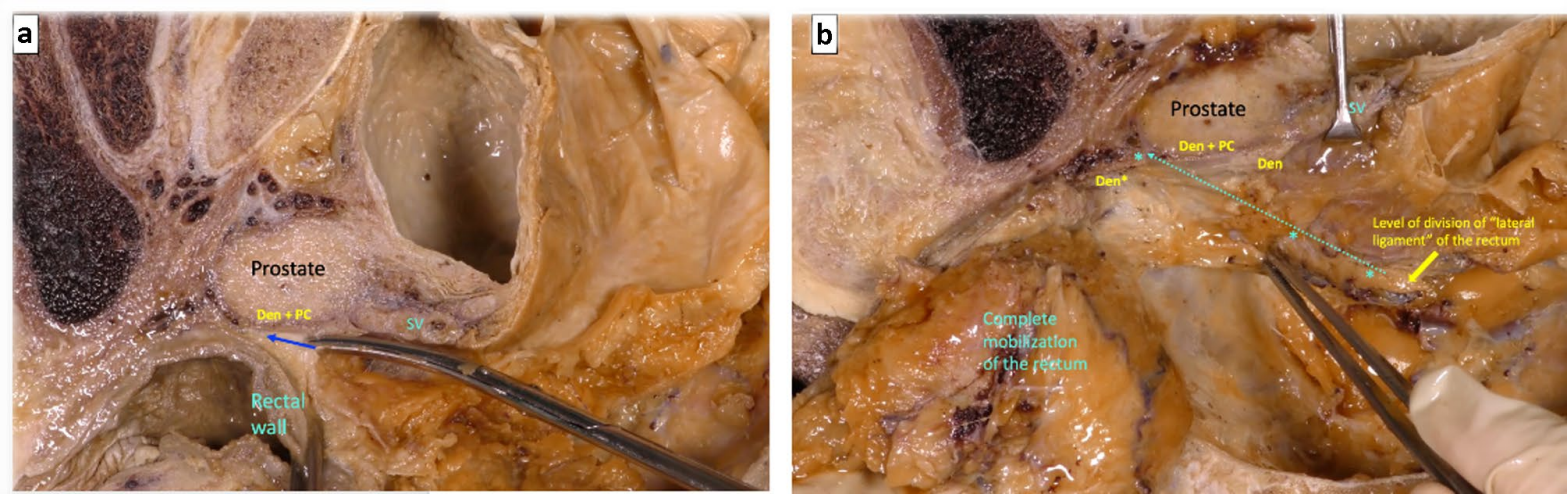
**a** After division of Denonvilliers’ fascia, there is single plane of loose areolar dissection (blue arrow) behind Denonvilliers’ fascia, tightly attached to the prostate capsule (*Den + PC*). **b** Complete mobilization of the rectum with preservation of nerve branches (from the inferior hypogastric plexus) and middle rectal vessels branches covered by the parietal fascia, running laterally downward and anteriorly in front of Denonvilliers’ fascia (*Den*) to form the neurovascular bundle on their way to the urogenital structures (blue dotted arrow and asterisks). *Den + PC*: confluence of Denonvilliers’ fascia and prostate capsule. Den*: Denonvilliers’ fascia extends downward to reach the level of the urogenital diaphragm (colour figure online)

By sectioning the anterior peritoneal reflection in its most anterior part, the backward pressure of the rectum makes it easy to access the first possible plane of dissection that is the loose areolar tissue anterior of Denonvilliers’ fascia and behind the seminal vesicle (Fig. 4a). When this plane of dissection is continued caudally, the firm adhesion of the fascia to the prostate capsule forces to section it (Fig. 4b) to be able to advance distally and complete the anterior dissection of the mesorectum (Fig. 5a). However, at the beginning of the dissection, the peritoneal reflection can be sectioned in its most posterior part, and a second possible plane of anterior dissection can be started in the loose areolar tissue posterior this Denonvilliers’ fascia and in front of the anterior mesorectum, covered by its thin visceral fascia. Following this anatomical plane downward, the dissection of the anterior mesorectum, that is very thin in its most distal part, can be completed (Fig. 5b).

After the complete dissection of the lateral and anterior mesorectum, the nerve branches from the inferior hypogastric plexus and the middle rectal vessels can be visualized, covered by the parietal fascia, running laterally downward and anteriorly in front of Denonvilliers’ fascia to form the neurovascular bundle on their way to the urogenital structures (Fig. 5b).

**Fig. 6:**
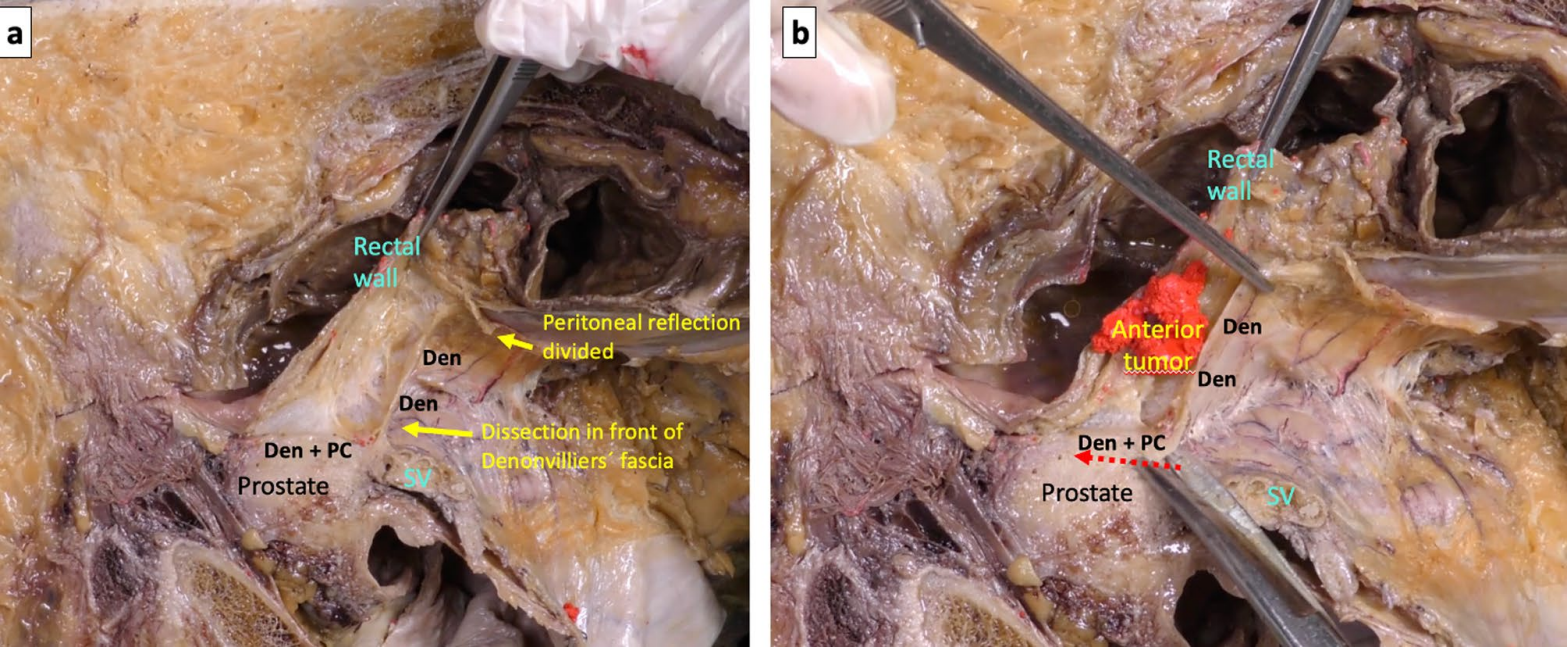
**a** The yellow arrow shows a dissection in front of Denonvilliers’ fascia (*Den*) and behind the seminal vesicles (*SV*) with a firm adherence of Denonvilliers’ fascia to the prostate capsule (*Den + PC*). **b** In the same specimen, with a supposed locally advanced anterior rectal tumor, the dissection is maintained in a non-anatomical plane (red dotted arrow) through a forced and sharp dissection including the confluence of Denonvilliers’ fascia and the prostate capsule (*Den + PC*) (colour figure online)

A third plane of anterior dissection can be described, which would be especially indicated in a locally advanced anterior and distal rectal tumor, which is very close or in contact with the area of firm adherence between Denonvilliers’ fascia and the prostate capsule. In this case, to avoid the risk of circumferential resection margin involvement, a non-anatomical plane can be used at this caudal level, through a forced and sharp dissection of the confluence area between Denonvilliers’ fascia and the prostate capsule, so that it would be included in the resected rectal specimen (Fig. 6). The potential planes of dissection for rectal cancer surgery, and the ideal indication for each of them, are summarized in Table 1.

**Table 1:** Potential planes of anterior mesorectal dissection with its indications in rectal cancer resection

1. *Anterior rectal tumors*: Dissection anterior and posterior to Denonvilliers’ fascia
(A) Cranial part of dissection in front of Denonvilliers’ fascia
(B) Section of Denonvilliers’ fascia previous to its adherence to the prostate capsule
(C) Distal part of dissection behind the confluence of Denonvilliers ‘fascia and prostate capsule
2. *Posterior rectal tumor*: Complete dissection posterior to Denonvilliers’ fascia
3. *Locally advanced anterior rectal tumors*
Complete dissection anterior to Denonvilliers’ fascia with excision of the prostate capsule

## Discussion

Knowledge of the anatomy and dissection planes is necessary to perform a proper and careful surgical procedure in pelvic surgery and more specifically in the treatment of rectal cancer, which could be reflected in lower local recurrence rates and improved results in terms of genitourinary function.

The current study clearly concurs with the description of the prostate-peritoneal membrane made by Denonvilliers in 1836, consisting of a separate fascial structure in the male cadaver pelvis, originated from the anterior peritoneal reflection, which descends initially approaching the seminal vesicle and subsequently adheres firmly to the prostate capsule in its entire extension. There is a clear clinical application of knowing in depth the anatomy of the Denonvilliers’ fascia; as demonstrated in the current cadaveric simulation model, different planes of dissection can be chosen, depending on the location of the rectal tumor.

Additional considerations are needed, when dealing with the anterior mesorectum in men. First, the anatomical descriptions of Denonvilliers’ fascia in the most current texts, mainly surgical, are not homogeneous, with different definitions in terms of its limits, structure, and composition. Its existence has been questioned in women, advocating for a clearer definition of the optimal plane of anterior dissection [2–5].

Several anatomical descriptions, such as those made by Testut [6], Netter [7] and Bannister [8], described the fascia as fused with the posterior surface of the prostate capsule, becoming part of it. Netter [7], described a variant that involves a division of the fascia into an anterior leaf that would merge with the prostate capsule, and a posterior one that would descend to the urogenital diaphragm. Likewise, most urological descriptions of the Denonvilliers’ fascia refer to it as intimately attached or fused to the prostate and seminal vesicles [9–13]. In a histological study, Kiyoshima et al. [9] found that, after radical prostatectomy without nerve preservation, Denonvilliers’ fascia was present in all specimens, and that in 97% of the cases, the fascia was fused to the prostate capsule in the central portion of the back of the gland.

The descriptions of the Denonvilliers’ fascia vary considerably among authors [3, 14]. According to Goligher [15], the fascia would be more firmly attached to the rectum than to the prostate, so it would be more convenient to separate it along with the rectum during rectal resection and divide it transversely below. Later, Heald et al*.* [16–18] described the Denonvilliers’ fascia as a trapezoidal “apron” of thickened anterior surface of the mesorectal envelope with a distinct and bloodless plane, with the seminal vesicles located in front of it. However, according to these authors, usually there is no clear surgical plane behind the Denonvilliers’ fascia, as this would be intimately adherent to the anterior mesorectal fat. Thus, they suggested that dissection posterior the Denonvilliers’ fascia might be technically more difficult and oncologically suboptimal. Distally, the Denonvilliers’ fascia becomes fused to the back of the prostatic capsule and the perineal body caudally. Therefore, it is necessary to section it to advance distally and complete the anterior dissection of the mesorectum.

Lindsey et al. [19] performed a histological study in surgical specimens from TME and described the Denonvilliers’ fascia as a structure that descends vertically from the bottom of the Douglas’ cul-de-sac to the pelvic floor, posterior and in proximity to the prostate and seminal glands, but without adhering to any of these structures.

The main controversy about Denonvilliers’ fascia topography is its firm adherence to the prostate capsule. The current cadaveric study showed that this fascia does not descend directly from the peritoneal reflection to the pelvic floor as a completely independent septum. In the current model, it was always firmly adhered to the prostate capsule without leaving a loose areolar plane of easy dissection between the two.

Such considerations have obvious consequences on the possible anatomical planes that can be generated during the anterior dissection of mesorectum. Dissection anterior to the Denonvilliers’ fascia achieves the best oncological outcome, whereas dissection posterior to the Denonvilliers’ fascia would help preservation of urinary and sexual function—without compromising the oncological outcome in selected patients.

Considering Denonvilliers’ fascia as an anterior independent septum without the need to section it to advance distally, Lindsey et al*.* [20] published a classification of the anterior dissection planes in rectal surgery, defining: (1) a peri-muscular plane, located just posterior to the rectum own fascia, (2) a mesorectal plane, located just anterior to the fascia that surrounds the mesorectum, offering an avascular plane for anatomical dissection, and (3) an extra-mesorectal plane, which is located just anterior to Denonvilliers’ fascia, leaving the seminal vesicles exposed when entering it. For non-anterior rectal tumors, they recommend the mesorectal plane anterior to the *visceral fascia of the mesorectum.*

In this sense, recently Fang et al*.* [21] came to similar conclusions. They carried out an anatomical study in four cadavers, focusing on the dissection of Denonvilliers’ fascia, which they described as originating in the peritoneal reflection and descending until reaching the perineal body independently of the fascia of the rectum. They specified that this fascia adheres more closely to the prostate than to the rectum. Therefore, dissection posterior to Denonvilliers’ fascia would be easier to perform than anterior to it. On the other hand, they described pelvic nerve fibers between the Denonvilliers’ fascia and the seminal vesicles, which led the authors to conclude that it might be prudent not to remove Denonvilliers’ fascia routinely during TME.

Adding to the controversy, in 2015, Kraima et al*.* [22, 23] published two histological studies on four human adult cadaveric specimens (two males and two females), and their findings were in keeping with the previous results of Heald et al*.* [18]. In their conclusions, Denonvilliers’ fascia is adherent to and continuous with the visceral mesorectal fascia like a multi-layered perirectal fascia, and therefore the optimal oncological plane during TME would be anterior to Denonvilliers’ fascia.

On the contrary, in 2019, Xu et al*.* [24] studied the pre-rectal space between the anterior rectal wall and the posterior surface of the prostate and seminal vesicles in six male cadavers, using sheet plastination examined under a confocal laser scanning microscopy. They identified several membrane-like structures within the pre-rectal space, with fibers originating from the external urethral sphincter and from the longitudinal rectal muscle and the connective sheaths of the neurovascular bundles. These would contribute to a multilayered membranous structure rather than a single fascial layer (as described by Denonvilliers) and confirmed that the optimal anterolateral plane for rectal mobilization would be posterior to this multilayered Denonvilliers’ fascia.

Similarly, Ghareeb et al*.* [25] recently supported the multilayer theory of Denonvilliers’ fascia in their anatomical study in 18 cadavers (13 male and 5 female). In male pelvis, they recommended a plane of dissection posterior to the first layer to preserve the multi-layered Denonvilliers’ fascia, commencing the anterior rectal mobilization by incising the peritoneum posterior to its reflection. The findings of the above-mentioned studies are summarized in Table 2.

**Table 2:** Summary of the articles with anatomical study of the pelvises

Author	Year	N pelvises	Topography of Denonvilliers’ fascia	Anatomical planes in relation to Denonvilliers’ fascia
Fang [20]	2019	4 (M)	An independent septum Nonadherence to prostate	Posterior to DF: The easiest Anterior to DF: Better oncological outcomes
Kraima [22, 23]	2015	4 (2 M and 2 W)	Adherence to visceral fascia of mesorectum Also in women	Anterior to DF
Xu [24]	2018	6 (M)	Multilayered structure from the external urethral sphincter to the longitudinal rectal muscle	Posterior to (preserving) DF
Ghareeb [25]	2019	18 (13 M and 5 W)	Supports multilayered theory	Posterior to (preserving) DF
Current study	2021	31 (M)	Adherence to prostate capsule Need to section it to advance distally	Anterior to DF for anterior tumors Posterior to DF for posterior tumors

M: men

W: women

DF: Denonvilliers’ fascia

The cadaveric simulation of the complete mobilization of the rectum performed in the current study, is in agreement with what Heald et al*.* [18] reported, meaning that the backward pressure on the rectum after the section of the anterior peritoneal reflection would make it be more natural or easier to enter the plane anterior to the Denonvilliers’ fascia. Staying in this loose areolar plane at the cranial part of the dissection is safe. The neurovascular bundles are located anterolaterally to the dissection plane of the anterior mesorectum: dissection in this anterolateral plane theoretically entails a possible risk of injury to these nerve branches, but, as reported by Heald et al*.* [17], and demonstrated with the current model, autonomic nerves can be preserved mastering the technique and with careful identification during the anterolateral dissection of the mesorectum.

Thus, the findings of the current study suggest that for an anterior tumor the ideal dissection plane is anterior to the Denonvilliers’ fascia to obtain the best oncological outcome. An important and technically more demanding example may be represented by locally advanced anterior tumors with possible contact with the prostate capsule. In this situation, to avoid the risk of affected circumferential resection margin, dissection should be along a plane anterior to Denonvilliers’ fascia, taking into account that entering this plane would force a sharp dissection of the fibrous structure caused by the adhesion between the Denonvilliers’ fascia and the prostate capsule.

However, the cadaveric simulation model also showed that the cranial plane behind the Denonvilliers’ fascia is accessible and represents a bloodless plane. These findings suggest, therefore, that for non-anterior rectal tumors an optimal oncological rectal dissection can be performed on the plane posterior to Denonvilliers’ fascia, with the aim of preventing urogenital dysfunction (Lindsey et al*.* [20], Huang et al. [26]). In this sense, Tejedor et al. [27] suggested that robotic surgery eases anterior dissection of the rectum preserving Denonvilliers’ fascia and Fang et al. [28] also give important surgical landmarks to identify the Denonvilliers’ fascia during TME as a white, thickened line at the peritoneal reflection, and that the dissection below this line helps the posterior entry to the Denonvilliers’ fascia.

## Conclusion

The variability of the reported descriptions generates a logical controversy. The fact that many studies are not based on pure anatomical investigations biases the transmission of the anatomical concepts. The cause of these differences could be the possible anatomical variability in the adult pelvis. However, the experience of many years in pelvic anatomical dissection of the authors of the current study, suggests the possible existence of dissection artifacts, which may be conditioned by previous judgments or prior knowledge, especially in the case of pelvises that are difficult to dissect. For this reason, the current study only included male cadavers that did not present pathological conditions, previous surgical traumas, or difficulties in the dissection due to anatomical abnormalities or a non-optimal conservation of the tissues after embalming and handling.

In summary, the current anatomical study defines the Denonvilliers’ fascia according to the original description made by Denonvilliers: a single-layer fascia originated from the peritoneal reflection that descends and adheres firmly to the prostate capsule. There are two possible anatomical planes of loose areolar tissue that can be generated in the more cranial part of the dissection, i.e., anterior versus posterior to the Denonvilliers’ fascia. In case of dissection anterior to it, the fascia needs to be sectioned to be able to complete the anterior mesorectal dissection distally.

The current cadaveric simulation model offers the possibility of choosing the most suitable plane for the anterior dissection of the mesorectum, depending on each individual case. Thus, the exact description of the Denonvilliers’ fascia and the dissection planes generated by it may have implications both on the surgical technique and on the oncological results, which could be reflected in a lower local recurrence as in a decrease in morbidity due to lesion of the pelvic autonomic innervation.

## References

[1] Denonvilliers C (1836). Anatomie du perinée. Bull Soc Anat Paris.

[2] Van Ophoven A, Roth S (1997). The anatomy and embryological origins of the fascia of Denonvilliers: a medico-historical debate. J Urol.

[3] Chapuis PH, Kaw A, Zhang M, Sinclair G, Bokey L (2016). Rectal mobilization: the place of Denonvilliers’ fascia and inconsistencies in the literature. Colorectal Dis.

[4] Zhang M, Kaw A, Chapuis PH, Bokey L (2016). Does Denonvilliers’ fascia exist in women?. Am J Obstet Gynecol.

[5] García-Gausí M, García-Armengol J, Mulas Fernández C (2021). Surgical anatomy of the rectovaginal space: does a standalone rectovaginal septum or denonvilliers fascia exist in women?. Dis Colon Rectum.

[6] Testut L, Latarjet A (1960). Tratado de Anatomía Humana. Tomo cuarto: Aparato de la digestión, peritoneo, aparato urogenital.

[7] Netter F (1982). Sistema digestive. Parte 2. Conducto inferior.

[8] Bannister LH, Williams PL (1998). Sistema Digestivo. Anatomía de Gray.

[9] Kiyoshima K, Yokomizo A, Yoshida T (2004). Anatomical features of periprostatic tissue and its surroundings: a histological analysis of 79 radical retropubic prostatectomy specimens. Jpn J Clin Oncol.

[10] Tewari A, Peabody JO, Fischer M (2003). An operative and anatomic study to help in nerve sparing during laparoscopic and robotic radical prostatectomy. Eur Urol.

[11] Brooks JD, Wein AJ (2007). Anatomy of the lower urinary tract and male genitalia. Campbell-walsh urology.

[12] Huland H, Noldus J (1999). An easy and safe approach to separating Denonvilliers’ fascia from rectum during radical retropubic prostatectomy. J Urol.

[13] Raychaudhuri B, Cahill D (2008). Pelvic fasciae in urology. Ann R Coll Surg Engl.

[14] Zhu XM, Yu GY, Zheng NX, Liu HM, Gong HF, Lou Z (2020). Review of Denonvilliers’ fascia: the controversies and consensuses. Gastroenterol Rep.

[15] Goligher J, Duthie H, Golinger J (1984). Surgical anatomy and physiology of the colon, rectum and anus. Surgery of the anus, rectum and colon.

[16] Heald RJ (1988). The ‘holy plane’ of rectal surgery. J R Soc Med.

[17] Heald RJ, Moran BJ (1998). Embriology and anatomy of the rectum. Semin Surg Oncol.

[18] Heald RJ, Moran BJ, Brown G, Daniels IR (2004). Optimal total mesorectal excision for rectal cancer is by dissection in front of Denonvilliers’ fascia. Br J Surg.

[19] Lindsey I, Guy RJ, Warren BF, Mortensen NJ (2000). Anatomy of Denonvilliers’ fascia and pelvic nerves, impotence, and implications for the colorectal surgeon. Br J Surg.

[20] Lindsey I, Warren BF, Mortensen NJ (2005). Denonvilliers’ fascia lies anterior to the fascia propia and rectal dissection plane in total mesorectal excision. Dis Colon Rectum.

[21] Fang J, Zheng Z, Wei H (2019). Reconsideration of the anterior surgical plane of total mesorectal excision for rectal cancer. Dis Colon Rectum.

[22] Kraima AC, West NP, Treanor D (2015). Whole mount microscopic sections reveal that Denonvilliers’ fascia is one entity and adherent to the mesorectal fascia; implications for the anterior plane in total mesorectal excision?. Eur J Surg Oncol.

[23] Kraima AC, West NP, Treanor D (2015). Understanding the surgical pitfalls in total mesorectal excision: investigating the histology of the perirectal fascia and the pelvic autonomic nerves. Eur J Surg Oncol.

[24] Xu Z, Chapuis PH, Bokey L, Zhang M (2018). Denonvilliers’ fascia in men: a sheet plastination and confocal microscopy study of the prerectal space and the presence of an optimal anterior plane when mobilizing the rectum for cancer. Colorectal Dis.

[25] Ghareeb WM, Wang X, Chi P, Wang W (2019). The “multilayer” theory of Denonvilliers’ fascia: anatomical dissection of cadavers with the aim to improve neurovascular bundle preservation during rectal mobilization. Colorectal Dis.

[26] Huang J, Liu J, Fang J, Zeng Z, Wei B, Chen T (2020). Identification of the surgical indication line for the Denonvilliers’ fascia and its anatomy in patients with rectal cancer. Cancer Commun.

[27] Tejedor P, Sagias F, Khan J (2020). Surgical anterior plane for rectal surgeons: preserving Denonvilliers’ fascia. Tech Coloproctol.

[28] Fang J, Huang J, Zheng Z, Wei B, Liu J, Huang Y (2019). How to find Denonvilliers’ fascia during laparoscopic TME. Tech Coloproctol.

